# Implementation of screening, brief intervention, and referral to treatment (SBIRT) in the emergency department without additional resources

**DOI:** 10.1186/1940-0640-8-S1-A54

**Published:** 2013-09-04

**Authors:** Janice L Pringle, Sherry Rickard-Aasen, Tamara Slain, Arvind Venkat

**Affiliations:** 1 University of Pittsburgh, School of Pharmacy and Therapeutics, Pittsburgh, PA, USA; 2 Allegheny General Hospital, Department of Emergency Medicine, Pittsburgh, PA, USA

## 

Patients who misuse substances present in inordinate numbers for emergency department (ED) services. Therefore, EDs are an important environment for identifying, intervening and connecting patients with treatment and recovery support to improve patient health and reduce healthcare utilization. EDs have largely depended on external funding or additional personnel to execute SBIRT. Our aim was to integrate SBIRT into the ED workflow and coordinate transfer to treatment and community support programs without added monetary/staff resources. Beginning in 2010, we worked cooperatively with a local ED to integrate SBIRT into the normal ED workflow. This program of screening, brief interventions and warm-handoff referral is dubbed “Safe Landing.” Efforts have focused on: training staff; embedding SBIRT tools into existing data systems; nurturing relationships with community treatment and recovery providers; developing protocols for a “warm-handoff” that would ensure patients who, in the context of a health crisis, express an immediate interest in following a road to recovery; and securing reimbursement for services. Over one-and-a-half years since implementation, 45,770 patients have been screened, with 7,996 assessed, 2,058 receiving a brief intervention, and 137 referred to treatment or recovery support. Multiple staff trainings have resulted in a palpable culture shift to patient advocacy and increasing compliance with SBIRT protocols. Screening and BI tracking tools embedded in the ED data systems continue to be enhanced. ED reimbursement for SBIRT began 10/2012, and cooperative relationships with treatment and recovery providers have diversified. We will discuss the implementation strategies employed to overcome challenges in operationalizing SBIRT in the ED. Challenges to be discussed include changes in key personnel, embedding SBIRT into the labyrinth of data systems, initial staff scepticism and evolving area treatment and recovery services organizations. Regardless, Safe Landing perseveres, and commitment by the local ED is stronger than ever.

